# A Multilevel Analysis of Neighborhood Socioeconomic Effect on Preterm Births in Georgia, USA

**DOI:** 10.3934/publichealth.2015.4.638

**Published:** 2015-09-22

**Authors:** Wei Tu, Jun Tu, Stuart Tedders

**Affiliations:** 1 Department of Geology and Geography, Georgia Southern University, Statesboro, Georgia 30460-8149, USA; 2 Department of Geography and Anthropology, Kennesaw State University, Kennesaw, Georgia 30144-5591, USA; 3 Jiann-Ping Hsu College of Public Health, Georgia Southern University, Statesboro, Georgia 30460-8015, USA

**Keywords:** Preterm Births, Multilevel Logistic Regression Models, Health Geography, Georgia, USA

## Abstract

This study estimates the neighborhood socioeconomic status (SES) effect on the risk of preterm birth (PTB) using multilevel regression (MLR) models. Birth data retrieved from year 2000 and 2010 Georgia Vital Records were linked to their respective census tracts. Principle component analysis (PCA) was performed on nine selected census variables and the first two principal components (Fac1 and Fac2) were used to represent the neighborhood-level SES in the MLR models. Two-level random intercept MLR models were specified using 122,744 and 112,578 live and singleton births at the individual level and 1613 and 1952 census tracts at the neighborhood level, for 2000 and 2010, respectively. After adjustment for individual level factors, Fac1, which represents disadvantaged SES, respectively generated an Odds Ratio of 1.056 (95% CI: 1.031–1.081) and 1.080 (95% CI: 1.056–1.105) for these two years, showing a modest but statistically significant effect on PTB. After adjusting for individual level factors and the census tract level factors, Intra-class correlation (ICC) was 1.2% and 1.4%, for year 2000 and 2010, respectively. The two IOR-80% intervals, 0.73–1.52 (year 2000) and 0.73–1.59 (year 2010) suggest large unexplained between census tract variation. The Median Odds Ratio (MOR) value of 1.21(year 2000) and 1.23 (year 2010) revealed that the un-modeled neighborhood effect was smaller than two individual-level predictor variables, race, and tobacco use but larger than the fixed effect of census tract-level predicting variable, Fac1 and all the other individual level factors. Overall, better census tract level SES was found to have a modest protective effect for PTB risk and the effects of the two examined years were similar. Large unexplained between census tract heterogeneity warrants more sophisticated MLR models to further investigate the PTB risk factors and their interactions at both individual and neighborhood levels.

## 1. Introduction

A birth occurring before the 37th week of pregnancy is a preterm birth (PTB)^1^. PTB is a significant public health issue and is responsible for more than one third of all infant deaths in USA, more than any other single cause [1],[2]. PTB is also a primary contributor of infant morbidity and children's developmental disabilities[3]. In addition, PTB causes significant financial burdens to the impacted families and the society. The U.S. health system pays an estimated $26 billion each year for PTB-related health problems [2],[4].

The crude PTB rate was 11.4% (nearly 500,000 births) in the U.S. in 2013. This rate was more than 10% lower than the rate in 2006 (12.8%) but was still higher than that of 1995 (11%). In the state of Georgia, the PTB rates were 11.9%, 14.2%, and 12.7% in 1995, 2006, and 2013, respectively, showing a similar rate change trend to the nation. Although PTB rates have been decreasing since 2006, both the U.S. and Georgia rates in 2013 were still much higher than the Healthy People 2020 target rate of 9.6% [5],[6].

Previous studies have established several individual-level PTB risk factors including a history of PTB, mother's socioeconomic status (SES, e.g., income), demographic (e.g., race) and behavioral (e.g., smoking during pregnancy) characteristics, and exposure to pollution [7]–[14].

Recognizing the hierarchical nesting of people within places and the importance of integrating individual risk factor epidemiology and ecological approach in health research, there has been a growing interest in studying the neighborhood effect (or contextual effect) on health outcomes. Pickett and Pearl [15], for instance, reviewed 25 published health studies with diverse research designs, health outcomes, and neighborhood measures. After controlling individual level predicting variables, the authors found statistically significant but generally modest association between social environment measures and health outcomes in 23 of the 25 studies.

Modest but significant association between adverse birth outcomes and neighborhood-level SES has also been reported in the literature. For instance, Herrick [16] found significant association between higher PTB (prior to 33 weeks' gestation) risk of urban black mothers and residing in low income neighborhoods in a North Carolina study; Roberts [17] reported that lower birth weight was associated with higher level of community poverty in a Chicago study. Kaufman et al. [18] found that living in wealthier neighborhoods would reduce the PTB risk. DeFranco et al. [19] in a study conducted in Missouri concluded that higher county-level poverty was associated with an elevated PTB risk. In a Baltimore study, O'Campo et al. [20] found that prenatal care had stronger protective effect on low birth weight risk for mothers living in neighborhoods with lower unemployment rates.

Further, multilevel models have been the most common type of analytical methods for assessing the effects of neighborhood residential environments on health outcomes [21], [22]. One notable advantage of this method is to allow proportioning the outcome variation at individual and neighborhood levels [23], [24].

Because efforts to predict PTB, as well as efforts to prevent it, have not received the expected success [25], prevention remains the key to reducing PTB risk [23], [24]. In addition, an early study by the U.S. Public Health Service reported that environmental factors and accessibility to health care contributed to roughly 30% of premature mortality [26]. Thus, a deeper understanding of the impacts of the social structure and neighborhood ecology on adverse birth outcomes can help the design of neighborhood-level prevention and intervention strategies to target high-risk geographical regions, facilitate allocation of resources for efficient local intervention, and track progresses toward Healthy People 2020 goals.

Our literature review identified several gaps in the neighborhood effect on adverse birth outcomes research. First, empirical studies focusing on poorly performed U.S. southeast including Georgia are limited in quantity, geographic coverage, and data availability. In Georgia, Ren [27] found that the residential instability had been associated with an elevated PTB risk. Messina [28] reported a statistically significant positive association between PTB risk and violent crime. But both studies were conducted in the city of Atlanta. A recent study at the state level showed that a higher census tract level SES would have a modest protective effect for PTB risk but the birth and census data were from year 2000 [29]. Thus, updated results based on data from more recent years are critical in understanding of the neighborhood effect over time.

Second, a range of individual census variables were selected as surrogates of neighborhood-level SES between the studies. These census variables varied from household income, poverty level, crime rate, to education, to name a few [17], [20], [30]. Each individual measure could capture at best one of many dimensions of the neighborhood SES, and the considerable variation in the SES measure make it very difficult to compare the results across studies. This difficulty may be overcome by applying composite variables based on multiple SES measures using Principal Component Analysis (PCA). PCA is generally employed to convert multiple potentially correlated variables into a set of uncorrelated variables that capture the variability in the underlying data, and it can also reduce the dimensionality of a dataset while attempting to preserve the relationships present in the original data [31].

Third, one often overlooked challenge in the application of MLR has been the effective interpretation of the neighborhood effect for models with binary outcomes. Although several statistical measures including Median Odds Ratio (MOR) and Interval Odds Ratio (IOR-80%) have been developed and have proven to be effective in many health studies [32],[33], these measures have just began to be introduced in adverse birth outcomes studies [29].

Aiming to fill these three gaps, this study is a substantive application and interpretation of MLR models using 2000 and 2010 Georgia birth data and census data. We converted nine census variables to uncorrelated variables (components) using PCA. The first two principal components were then used in the MLR models to represent the neighborhood SES. We calculated and used Median Odds Ratio (MOR) and Interval Odds Ratio (IOR-80%) to interpret the neighborhood effect on PTB risk of our models.

There are three major objectives in this empirical study: 1) estimate and partition the variance of PTB risk at individual and census tract levels; 2) estimate the effect of census tract level SES on PTB risk after controlling individual characteristics; and 3) compare the models results between 2000 and 2010 to confirm the findings of the effect of SES on PBT risk

## 2. Data and Methods

### 2.1. Individual-and neighborhood-level variables

The birth data of 2000 and 2010 were collected from the electronic birth certificate data from Georgia Department of Public Health. The mothers' self-reported residential addresses were geocoded as the locations of the births and these locations were linked to the census tracts. Only live and singleton births with complete individual and census tract level census were included in the analyses.

The model outcome, PTB, is a binary variable. A value of one “1” are for births before completing 37 weeks of pregnancy and a value of zero “0” are for births on or after 37 weeks of pregnancy. Six individual-level predicting variables were included in the models: race (black, white, others), sex (male or female), age (mother's age in years), marital status (married or unmarried), education (mother received less than nine years of education, yes or no), and smoking during pregnancy (yes or no). We selected these variables because they had been considered well-established risk factors according to two authoritative premature births studies [4], [23].

To develop a standardized neighborhood level SES in multilevel models to allow results from different studies more comparable and replicable, Messer et al. [31] selected eight census variables from 20 census variables after conducting a comprehensive literature review of socioeconomic and demographic factors associated with health outcomes. These variables were the percent of males in management and professional occupations, percent of crowded housing, percent of households in poverty, percent of female headed households with dependents, percent of households on public assistance and households earning < $30,000 per year, percent less than a high school education, and the percent unemployed [31]. The first principal component resulting from PCA analysis of the eight variables was used as a proxy of neighborhood level SES measure, the deprivation index. This index was found to be associated with the unadjusted prevalence of PTB and low birth weight births for white non-Hispanic and black non-Hispanic women in the eight study areas across the states of Maryland, Michigan, North Carolina, and Pennsylvania.

In this study, we followed Messer et al.'s approach with minor adjustment in the selection of census variables. We selected nine instead of eight census variables including poverty, female household head, household income < $25,000, occupation in management sectors, unemployment, percent population receiving public assistance, average household size, vehicle ownership, and population receiving less than high school education. We added one more census variable, vehicle ownership, because we believe that mothers' mobility is an important aspect of the overall neighborhood SES that should be considered in the analyses. In addition, we used household income < $25,000 instead of < $30,000 (Table 1).

**Table 1:** Individual-and Neighborhood--Level descriptors Based on Georgia Vital Records and Census Data

Variable	Method of Computation/Unit	(% for binary variables with the value of 1) Mean ± SD for continuous variables	
		2000	2010
Individual characteristics (2000: N=122,744; 2010: N=112,578)			
Gestation Weeks	<37 weeks = 1; >=37 weeks = 0	11.15%	13.79%
Race/ethnicity	White =1; Black =2; Others =3	62.46%	54.98%
Sex of the newborn baby	Male =1; Female =0	50.40%	51.07%
Mother's age		26.48 ± 6.13	27.13 ± 6.15
Mother's Marital Status	Married = 1; Unmarried = 2	62.55%	55.23%
Mother ‘s had less than 9 year ofeducation	Yes =1; No =0	5.44%	2.32%
Mother Used Tobacco DuringPregnancy	Yes =1; No =0	8.33%	7.08%
Neighborhood (Census Tract) characteristics (2000: N = 1,613; 2010: N = 1952)			
Poverty	% population living below federalpoverty	15.91±12.29	19.40±13.03
Household income	% households with income less than$25,000	33.06±17.23	28.20±15.60
Female household head	% families with female headedhousehold with dependent children	9.24±6.54	10.86±7.23
Public Assistance	% households receiving publicassistance	3.58±3.67	1.85±1.92
Occupation	% in management	29.41±13.34	33.30±14.43
Household size	Average household size	2.63±0.34	2.68±0.40
Unemployment	% unemployed population	3.93±3.93	11.21±5.90
Education	% population with no high schooleducation	23.30±12.50	16.74±10.46
Vehicle ownership	% households with no (owned orrented) vehicle	10.43±11.56	8.05±9.12

PCA was performed on the nine selected variables on census-tract level for year 2000 and 2010. Year 2000 data were collected from US Census 2000. Because 2010 US Census no more provides these variables, we used American Community Survey (ACS) 5-year (2008–2012) estimates to represent the 2010 data. PCA without rotation for both years were run. The first two principal components (factors) had eigenvalues larger than 1.0 were retained and then used as neighborhood level SES measures in the regression models later.

### 2.2. Selection of Neighborhood

We chose census tracts to approximate neighborhoods in our models. Census tracts are relatively small and stable statistical geographic units with fairly homogenous SES and living conditions, containing on average 4,000 residents. Although we are fully aware of the potential drawbacks of using census tract as proxy of neighborhoods, we also believe that census tracts allow convenient and consistent data collection and have been considered as at least an acceptable approximation of a person's immediate residential environment in health studies literature [18], [30], [34], [35].

### 2.3. Statistical Analysis

#### 2.3.1. Model Specification

The multilevel modeling approach brings individual risk factor epidemiology and an ecological approach into one analytical framework. Multilevel models produce association conventional measures (in the format of regression coefficients, odds ratios etc.) of ordinary regression models. In addition, they estimate variance partition between individual and neighborhood levels for understanding the relative importance of predicting variables to health outcomes at different levels [36]. As such, multilevel modeling remains to be a dominating analytical method of studying neighbourhood socioeconomic context and health outcomes [37].

The multilevel logistic regression (MLR) models built in this study were all two level models in which individuals (mothers, level 1) were nested within neighborhoods (census tracts, level 2). The full multilevel model is described conceptually below and readers interested in the formal statistical notations and explanations can refer to other references [37], [38].

Birth outcome (yes or no) = baby's sex + mother's age-25 + mother's race/ethnicity + mother's marital status + mother's education + mother's tobacco use during pregnancy + census tract-level SES Factor 1 + census tract-level SES Factor 2 + random effects (at the census tract level).

The random part (level 1 and 2 variances) and the fixed part (regression coefficients) of the models were estimated using maximum likelihood with the Laplace approximation. For the purpose of comparison, we also fitted an ordinary logistical regression model that included all the individual level predicting variables and census tract level Fac1 and Fac2. All the models were developed and fitted using R v. 3.13 [39].

#### 2.3.2. Variance Partition and Model Interpretation

The intraclass correlation coefficient (ICC) is a variance partition coefficient. It can be calculated as the percentage of the neighborhood level variance in the total (both individual and neighborhood) variance. A high ICC value indicates that the outcome difference comes more from the difference in neighborhoods than in individuals [40]. In MLR, the individual level variance is a constant, 3.29. Thus, ICC is calculated as:

$$\text{ICC}=\frac{{\text{τ}}^{2}}{3.29+{\text{τ}}^{2}}$$ (1)

Where, τ^2^ is the neighborhood level variance.

Although ICC is a convenient and intuitive measure, its application on MLR models is problematic because variances at the two levels are on difference scale, the individual level is on a probability scale and the neighborhood level is on a logistic scale. Therefore, ICC may not accurately represent the partitioning of variance in MLR models. In addition, ICC has issues in its interpretation and generalizability [41].

The Median Odds Ratio (MOR) is introduced and used to overcome this limitation. It takes two steps to obtain MOR. First, a set of odds ratios is generated by comparing pairs of mothers with identical individual-level characteristics but from two randomly chosen, different neighborhoods (i.e., with different neighborhood random effect). Next, identify the median of this set of odds ratios, which is MOR. MOR can be understood as the median odds between two mothers having PTB, who are living in two neighborhoods with different PTB propensity. The value of MOR is always equal to or greater than 1. A MOR of 1 indicates zero between-neighborhood variation in PTB risk. The large MOR value, on the other hand, indicates higher between-neighborhood variation in PTB risk that is not explained by the modeled neighborhood-level predicting variables [42]. MOR is calculated in Equation 2 as a function of *τ*^2^, the variance of neighborhood effect:

$$\text{MOR}=\text{exp}[2\times \tau^{2}\times 0.6745]\approx \text{exp}(0.95\tau )$$ (2)

Where, *τ*^2^ is the variance of neighborhood effect.

Conventional interpretation of odds of individual-level predicting variables can also be applied in MLR models to compare individuals located within the same neighborhood. For example, a racial effect can be interpreted as the odd ratio of having a PTB between a white mother and a black mother who live in the same neighborhood and with the same individual predicting variables except for their race.

However, the interpretation of the results of neighborhood-level predicting variables of MLR models is much less straightforward. The odds ratio of the outcome is interpreted as comparing two neighborhoods with one unit difference in the value of the predicting variable but having the identical random effect. In the context of this research, for instance, the odds ratio of having a PTB is to comparing two mothers living in two census tracts with one-unit difference in factor value and with the identical random effect.

In this study, we introduce a statistic measure, 80% interval odds ratio (IOR-80%), to provide a more intuitive interpretation of neighborhood effect. IOR-80% is used because this measure incorporates both the fixed neighborhood-level predicting variable effect and the unexplained between-neighborhood heterogeneity in one single interval value [31], [42]. It also takes two steps to calculate IOR-80%. First, we calculate the odds ratio of all pairs of mothers with identical individual-level predicting variables from two neighborhoods with a one-unit difference in the value of the neighborhood-level predicting variable (i.e., Fac1 and Fac2). We then examine the distribution of all the calculated odds ratios. IOR- 80% is an interval that contains 80 % of the odds ratio values at the median. IOR-80% can be computed using Equation 3:

$$\text{IOR}-80{\text{\%}}_{\frac{\text{lower}}{\text{upper}}}=\text{exp(β}\pm 1.2816\sqrt{2\times {\text{τ}}^{2}})\approx \exp\left(\text{β}\pm 1.81\text{τ}\right)$$ (3)

Where, β is the regression coefficient of the neighborhood-level predicting variable, τ^2^ is the neighborhood-level variance, and -1.2816 and + 1.2816 are respectively the 10^th^ and the 90^th^ centiles of the standard normal distribution.

Equation 3 shows that a smaller between-neighborhood variation (τ^2^) will generate a narrower IOR-80%, whereas a larger between-neighborhood variation (τ^2^) will generate a wider IOR-80%. IOR-80% combines the measure of unexplained between-neighborhood variation and the effect of the neighborhood-level predicting variable included in the MLR model. In addition, IOR-80% should contain 1 if the value of τ^2^ is larger than the effect of the neighborhood-level predicting variable.

## 3. Results

### 3.1. Descriptive statistics

Table 1 shows the summary statistics of individual and census-tract level variables for 2000 and 2010. A total of 122,744 and 112,578 live and singleton births were included in this study for 2000 and 2010, respectively. The values of these variables between the two years were generally comparable. At the individual level, for 2000, the births with less than 37 gestation weeks, race of white, gender of male were 11.45%, 62.46%, and 50.4%, respectively, while for 2010, these variables were 13.79%, 54.98%, and 51.07%, respectively. For 2000, the average mother age was 26.48, compared to 27.13 for 2010. The mothers who were married, had less than 9 years of education, and used tobacco during pregnancy for 2000 account for 62.55%, 5.44%, and 8.33%, respectively, while the three variables for 2010 were 55.23%, 2.32%, and 7.08%.

The births were located in 1,613 census tracts in Georgia for 2000, and 1,952 for 2010. There are also some reasonable differences in the census-tract level variables between the two periods. On the average, for 2000, % population living below federal poverty line, % households with income less than $25,000, % families with female headed household with dependent children, and % households receiving public assistance were 15.91, 33.06, 9.24, and 3.58, respectively, while these four variables for 2010 were 19.4, 28.2, 10.86, and 1.85. The average household size for 2000 was 2.63, very close to 2.68 for 2010. Averagely, % unemployed population, % population with no high school education, and % households with no vehicle were 3.93, 23.3, and 10.43, respectively, for 2000, while these three variables for 2010 were 11.21, 16.74, and 8.05. As indicated by the Standard Deviations, significant variations exist in each of the 9 neighborhood-level variables among census tracts for both time periods.

### 3.2. PCA Results

Table 2 shows factor loadings and variance explained by factors by PCA. The first two principal components (factors) with eigenvalues larger than 1.0 were retained. They explained 76.39% and 66.86% of the total variance in the data for year 2000 and 2010, respectively. For both years, the first factor had high positive loadings on % Population Living below Federal Poverty, % Female Household Head, % Households with Income Less Than $25, 000, % Households Receiving Public Assistance Income, % Population with Less Than High School Education, % Unemployed Population, and % Households without Vehicles, but a high negative loading on % Population in Management Profession. Thus, factor 1 (Fac1) can be considered as disadvantaged SES measure. For both years, the second factor only had a positive heavy loading on Household Size, but with low loadings on other variables. So factor 2 (Fac2) can be used as a household size measure. These two factors were used as composite neighborhood level SES measures in the regression models.

**Table 2:** Factor Loadings and Variance Explained from PCA for both 2000 and 2010

Year		2000		2010	
Retained Factors		Factor 1	Factor 2	Factor 1	Factor 2
Factor loadings of Variables	Poverty	0.924	-0.171	0.884	-0.178
	Female household head	0.776	0.204	0.721	0.179
	Household income	0.917	-0.178	0.900	-0.287
	Public Assistance income	0.869	0.073	0.552	0.122
	Occupation	-0.699	-0.497	-0.749	-0.372
	Household size	-0.035	0.915	0.003	0.929
	Unemployment	0.732	-0.260	0.695	0.137
	Education	0.803	0.248	0.767	0.194
	Means of transportation	0.874	-0.257	0.769	-0.399
% of Variance Explained		60.96	15.43	51.55	15.32
Cumulative % of Variance Explained		76.39		66.86	

### 3.3. Results of MLR models

Three MLR models were fitted for 2000 and another three for 2010. For the three models for either year, M0 was a null model with no predicting variables, M1 included only individual level predicting variables, and M2 added two census tract level SES predicting variables, Fac1 and Fac2, to M1. The effect of Fac2 will not be discussed below because it was statistically insignificant.

Partial model results for the two years are shown in Tables 3–4. M2 estimated that the odds ratio of Fac1 was 1.056 and 1.080, for 2000 and 2010, respectively. If comparing two mothers with identical risk factors residing in two census tracts with one unit difference in Fac 1 and if the two census tracts were otherwise identical with regard to PTB risk, then the odds of having PTB for the mother residing in the census tract with the higher Fac1 value was 1.056-fold and 1.080-fold higher, for 2000 and 2010, respectively.

As discussed in section 2.3.2 of the paper, due to the statistical nature of MLR models, it is neither intuitive nor useful to directly interpret the odds ratio of the neighborhood effect. So IOR-80% is introduced to facilitate the interpretation. M2 estimated that the IOR-80% for Fac1 was 0.73 to 1.52 and 0.73 to 1.59, for 2000 and 2010, respectively. These two data intervals suggests that when comparing two randomly chosen mothers with identical individual level characteristics, one from a census tract with one unit higher Fac1 than the census tract the other was from, and the two census tracts possibly differing in other ways regarding PTB risk, the odds ratio for the comparison would, with 80% probability, lie between 0.73 to 1.52 and 0.73 to 1.59, for 2000 and 2010, respectively. The relatively wide IOR-80% intervals suggest substantial residual variation in PTB risk between census tracts and considerable uncertainty in the impact of census tract level Fac1 on PTB risk. In addition, this residual was neither accounted for by census tract level Fac1 nor by mothers' individual characteristics of the MRL models.

The odds ratios of Fac1 in the ordinary logistic regression (GLM) and M2 were very close and the effects were statistically significant in both models (*P* < 0.001). However, the 95% CIs for Fac1 in M2 was slightly wider than in the ordinary logistic regression, reflecting that the MLR model accounted for a small portion of between census tract heterogeneity.

**Table 3:** Modeling Results: 2000

	GLM		M0	M1		M2	
	OR	95%Cl	OR	OR	95%Cl	OR	95%Cl
Level 1-individual N=122,744							
Black	1.406	(1.35,1.47)		1.456	(1.39,1.52)	1.412	(1.35,1.48)
Other	0.934	(0.84,1.04)		0.948	(0.85,1.06)	0.947	(0.85,1.06)
Female	0.899	(0.87,0.93)		0.899	(0.87,0.93)	0.899	(0.87,0.93)
AGE25	1.014	(1.01,1.02)		1.013	(1.01,1.02)	1.014	(1.01,1.02)
Unmarried	1.206	(1.15,1.26)		1.222	(1.17,1.28)	1.206	(1.15,1.26)
Education < 9 years	1.008	(0.93,1.09)		1.038	(0.96,1.13)	1.027	(0.95,1.11)
Tobacco use	1.332	(1.25,1.42)		1.330	(1.25,1.41)	1.320	(1.24,1.4)
Level 2-census tract N = 1613							
Fac1	1.056	(1.034,1.078)				1.056	(1.031,1.081)
Fac2	0.987	(0.97,1.01)				0.990	(0.97,1.01)
IOR-80%-Fac1							(0.73,1.52)
Measures of census tractlevel variation							
MOR						1.213	
ICC			0.021		0.013	0.012	
Model Selection							
AIC	87124		87723	87070		87044	

M2 estimated that between census tracts variation contributed 1.2% and 1.4% to the total variance in PTB risk for 2000 and 2010, respectively. The low ICC value for PTB suggests much greater heterogeneity within census tracts (between individuals) than between census tracts. MOR is calculated to provide information on unexplained heterogeneity between census tracts. M2 estimated that MOR was 1.21 and 1.23 for 2000 and 2010, respectively. These two numbers can be interpreted as, if a mother moved from a census tract to another with a higher PTB propensity, the median increase in the odds of having PTB would be 1.21-fold and 1.23-fold for 2000 and 2010, respectively. These two numbers also indicated that the effect of unexplained between neighborhood variation on PTB risk was weaker than the effects of two individual-level predictor variables, race and tobacco use but larger than the fixed effect of census tract-level predicting variable, Fac1 and all the other individual-level predictor variables. The Akaike Information Criterion (AIC) value of M2 was 87044 and 89356, for 2000 and 2010, respectively, smaller than the respective values from the GLM, 87124 and 89442 in the two years, indicating that a multilevel was a better modeling choice than a non-multilevel one in this case [43].

**Table 4:** Modeling Results: 2010

	GLM		M0	M1		M2	
	OR	95%Cl	OR	OR	95%Cl	OR	95%Cl
Level 1-individual N= 112,578							
Black	1.367	(1.31,1.42)		1.429	(1.37,1.49)	1.372	(1.31,1.43)
Other	0.982	(0.91,1.05)		0.992	(0.92,1.07)	0.988	(0.92,1.06)
Female	0.973	(0.94,1.01)		0.972	(0.94,1.01)	0.972	(0.94,1.01)
AGE25	1.018	(1.02,1.02)		1.017	(1.01,1.02)	1.019	(1.02,1.02)
Unmarried	1.262	(1.21,1.32)		1.285	(1.23,1.34)	1.263	(1.21,1.32)
Education < 9 years	1.032	(0.92,1.16)		1.079	(0.96,1.21)	1.048	(0.94,1.18)
Tobacco use	1.238	(1.16,1.32)		1.245	(1.17,1.33)	1.230	(1.15,1.31)
Level 2-census tract N = 1952							
Fac1	1.080	(1.059,1.101)				1.080	(1.056,1.105)
Fac2	0.010	(0.99,1.03)				0.990	(0.99,1.03)
IOR-80%-Fac1							(0.73,1.59)
Measures of census tractlevel variation							
MOR						1.226	
ICC			0.023		0.015	0.014	
Model Selection							
AIC	89442		90105		89401	89356	

## 4. Discussions

Our modeling results indicate that the association between census tract level SES and PTB was weak but statistically significant and that the size of the variance among census tracts was modest compared with the variance among individuals. Our finding is overall consistent with the conclusions found in existing literature. In addition, the magnitude of the neighborhood effect on PTB risk in 2000 and 2010 was comparable.

The results of this study should be interpreted with caution. First, our analyses were based on vital records data, a secondary dataset with varying reliability and questionable quality. For instance, no individual level economic factor was controlled in our models due to the lack of data. Inadequate control of individual level SES can lead to overestimation of the effect of neighborhood level SES [44]. Due to the lack of data, mothers' respective mailing addresses were used to represent mothers' actual exposure time in the specific neighborhoods, an acceptable but imperfect treatment. Despite these problems, the best available data were utilized in our analyses when this study was conducted.

Second, as many previous studies, we chose to use census tract as proxies of neighborhoods allowing mainly for more convenient data collection and results comparison with other research. But artificial administrative boundaries designed for census data gathering and reporting may not be effective in capturing social and cultural customs, values, and perceptions that are important factors for defining actual neighborhood boundaries.

Third, a standardized neighborhood SES index (i.e., Fac1) was calculated to provide a comprehensive summary of neighborhood SES and to allow consistent comparison across studies in the U.S. One potential problem of the index approach is that it will not discriminate between the effects of individual neighborhood characteristics. Thus, a single SES measure may be more appropriate in modeling the impact of one particular type of neighborhood characteristics.

Fourth, there were different levels of changes in the selected census-tract level SES variables from 2000 to 2010 in this study. We used PCA factor scores derived from these variables in the MLR models. These factors were standardized, so they reflected only the relative neighborhood SES of the individual census tracts in the respective year, but not the changes of neighborhood SES from 2000 to 2010. It will be interesting to examine the change of neighborhood SES over the two time periods and to compare how this change may affect PTB risk, but we decided in this study to check only whether the effect of neighborhood SES on PTB risk is consistent in the two time periods using MLR models.

Fifth, statistically, MLR models depend on atypical individuals in the neighborhood to distinguish between individual level and neighborhood level effects. But atypical residents usually account for only a small population in a neighborhood in reality. For instance, black women living in predominantly white neighborhoods may be more vulnerable to PTB risk, but black women are more likely to live in predominantly black than white neighborhoods. So neighborhood effect on PTB needs to be considered together with PTB prevalence and relative risks because policy efforts focusing only the small number of atypical individuals would be inefficient.

A small neighborhood variance should not discourage us from further exploring the contextual effects on PTB risk. Statistically, our results show that there was considerable unexplained heterogeneity between census tracts. Substantively, consistent uneven geographic distribution of raw PTB rates has been observed in Georgia and in the U.S. in the past two decades, and physical and socioeconomic environment also varies significantly across different geographic regions across the state and the country.

We propose three improvements to advance the investigation of neighborhood level SES on adverse birth outcomes. First, model interactions between neighborhood SES and individual level risk factors to examine causal pathways between the neighborhood SES context and birth outcomes. Neighborhood level changes may affect downstream individual characteristics, which in turn influence individual health outcomes including PTB. A better measure of this chain of events can help our understanding and designing effective community-based intervention programs. For instance, a neighborhood-based tobacco free campaign may reduce a mother' smoking behavior, which in turn may lower the PTB risk of this mother.

Second, test two promising new approaches in addressing the uncertain geographic context problem [45]. One is to build and use larger analytical units from basic geographic areas (e.g., zip code areas). These larger units will have larger and more stable base population but still maintaining coherent areal SES characteristics and spatial closeness [46]. The other is to run sensitivity analysis to assess changes in modeling results in response to changes in contextual units [47].

Third, build MLR models to examine additional contextual factors including low accessibility of health care services and exposure to pollution and their cofounding effects with individual characteristics. Because mothers who reside in low SES neighborhoods are also likely to be exposed in these related contextual risk factors of PTB.

As is the case in most studies regarding complex public health issues involving SES and health outcomes, a clear causal mechanism has yet to be established between the social construct of neighborhood and birth outcomes. Some studies have suggested that neighborhood-level SES might operate as a proxy for unmeasured individual characteristics [48], [49]. However, based on a review of the literature, we tend to believe that neighborhood-level SES influences health outcomes over and above individual SES. In fact, there are several possible ways neighborhood SES can influence the health of pregnant women. Neighborhood SES, particularly as it relates to the basic necessities of life, may influence the level of stress a woman experiences during her pregnancy [50]. Moreover, there is a strong association between cultural norms and the SES of a given neighborhood. Thus, SES may also impact a woman's decision on the use of hazardous substances during pregnancy such as tobacco, narcotics, or alcohol [51]. Research has also shown that prenatal exposure to air and water pollutants may have a detrimental effect on fetal development [14], [52].

Additional influences include the fact that neighborhood SES impacts the availability and access to critical prenatal care and other health services vital to the health of the baby [53], as well as influencing accessibility of proper nutrition critical to fetal development [54]. Furthermore, it is hypothesized that structural and contextual factors may modify health outcomes by interacting with individual factors related to life style and behaviors to modify biological processes [55]. A thorough and more theoretical-based investigation on the potential direct and mediated pathways through which neighborhood-level SESE on adverse birth outcomes is beyond the scope of this study. Interested readers may find discussions elsewhere in the literature [56]–[58].

## 5. Conclusions

In this study, we constructed two-level MLR models to estimate the impacts of neighborhood SES on PTB using vital records data and census data in Georgia in 2000 and 2010. We computed a standardized neighborhood SES index using PCA and applied the index in our MLR models. We calculated two statistic measures to facilitate the interpretation of the modeled and un-modeled neighborhood effects on PTB.

Between census tracts accounted for 1.2% and 1.4% of the total variance for 2000 and 2010, respectively, after adjusting the individual factors and census tract level SES. The fixed census tract-level SES effect, Fac1, was 1.056 and 1.080, for 2000 and 2010, respectively, showing a weak but significant relationship between low neighborhood SES and elevated PTB risk. In other words, higher census tract level SES served as small protective factor to PTB risk. The two MOR values, 1.21 and 1.23, suggests that unexplained heterogeneity between census tracts should not be ignored in understanding the PTB risk. The two relatively wide IOR-80% intervals, 0.73 to 1.52 (2000) and 0.73 to 1.59 (2010), further confirms substantial between census tracts residual variation in PTB risk and considerable uncertainty of the census tract level SES effect on PTB risk.

In summary, we have made three contributions in advancing birth outcome studies using multilevel analyses. First, we examined and compared the neighborhood SES effect on PTB using MLR models in 2000 and 2010. Most of the previous models used either data of one particular year or the average data of multiple years, which provided only a static snapshot of the neighborhood effect. To provide useful information to guide public health policy, it is necessary to routinely updating models with recent health and neighborhood data. Second, the PCA components derived from this study can be further tested and developed to construct a measure that can best represent the real neighborhood SES. Last but not least, the introduction of MOR and IOR-80% helped the interpretation of otherwise unintuitive results from MLR.

Due to its intrinsically nested hierarchical data structure [59], [60], health outcomes research is vulnerable to the “ecological fallacy [61], transferring observations at an aggregate level to an individual level outcome, and to the “atomistic fallacy” [62], ignoring the socioeconomic context that may alter an individual level outcome. A MLR approach offers an effective methodological framework to deal with these challenges and to help identify causal inference of health outcomes. More sophisticated modeling strategies (e.g., developing more silent analytical units, modeling both intercept and slope or interaction between predicting variables at different levels) should be developed to provide more clues of the neighborhood effect on adverse birth outcomes [37]. The ultimate goal of these modeling effects is to target geographical areas for resource allocation and to formulate prevention and intervention programs that will most effectively reduce the PTB risk.

## Footnotes

1 Abbreviations: PTB, preterm birth; MLR, multilevel logistic regression; SES, socioeconomic status; MOR, median odds ratio; IOR-80%, 80% interval odds ratio; ICC, intraclass correlation coefficient; AIC, Akaike information criterion; PVC, proportional variance change.
